# A Rare Case of Isolated Ovarian Tuberculosis Incidentally Found During In Vitro Fertilization Workup in a Young Woman With Primary Infertility

**DOI:** 10.7759/cureus.27051

**Published:** 2022-07-20

**Authors:** Meriem Rhazari, Afaf Thouil, Hatim Kouismi, Jamal eddine El Bourkadi

**Affiliations:** 1 Department of Pulmonology, Mohammed VI University Hospital, Oujda, MAR; 2 Department of Pulmonology, Faculty of Medicine and Pharmacy, Mohammed VI University Hospital, Mohammed I University, Oujda, MAR; 3 Respiratory and Allergic Diseases, Mohammed VI University Hospital, Oujda, MAR; 4 Department of Pulmonology, Moulay Youssef Hospital, Rabat, MAR

**Keywords:** salpingitis, ovarian tuberculosis, extrapulmonary tuberculosis (eptb), infertility, genital tuberculosis

## Abstract

Female genital tuberculosis (TB) is a common form of extrapulmonary TB (EPTB). It is a major cause of infertility in low-income countries, and in many cases, it is asymptomatic and typically not diagnosed until the patient seeks medical advice for infertility. The infection is usually secondary to primary pulmonary TB via hematogenous or lymphatic dissemination, but sexual transmission through genital TB of the partner is also possible. We describe a rare case of isolated ovarian TB as a fortuitous diagnosis during in vitro fertilization (IVF) workup in a 40-year-old woman with primary sterility and no specific symptoms. This case highlights the importance of screening for TB before an IVF procedure in women with infertility, especially in countries with a high prevalence of this disease.

## Introduction

Tuberculosis (TB) is a chronic infectious disease commonly caused by Mycobacterium tuberculosis (99% of the cases) [1]. TB affects millions of individuals worldwide and over one million people die from TB each year [2]. The infection mainly affects the lungs, but extrapulmonary involvements are also common [2]. The lymph node cases of TB represent the most frequent forms of extrapulmonary TB (EPTB), followed by urogenital TB (UGTB), with an estimated prevalence of 30-40% among all EPTB cases [2]. UGTB usually results from hematogenous or lymphatic dissemination of the pathogen from a primary infected lung or other organs, but sexual transmission through a partner with genital TB has also been described [2,3]. In women, the most common site of genital TB is the fallopian tubes [4].

Female genital TB is usually asymptomatic and is often diagnosed during diagnostic workup for infertility [5]. However, patients with this condition may present with low-grade fever, weight loss, chronic pelvic pain, menstrual irregularities, vaginal bleeding, secondary amenorrhea, or a pelvic mass [4,6]. Its diagnosis is often delayed, and it is frequently misdiagnosed as ovarian cancer due to the varying nature of its clinical presentations [2,4]. The definitive diagnosis of TB requires bacteriological evidence via culture on Löwenstein-Jensen (LJ) medium or by acid-fast staining with the Ziehl-Neelsen technique. However, histological evidence is usually accepted in UGTB due to the paucibacillary forms in this entity [1,2,7]. We present a rare case of isolated ovarian TB in a 40-year-old woman with four years of infertility, incidentally diagnosed by laparoscopy during in vitro fertilization (IVF) workup.

## Case presentation

A 40-year-old married Moroccan woman with a four-year history of primary sterility secondary to a bilateral cystic ovarian endometriosis presented for an IVF workup. She had benefited from several cystectomies with a left salpingectomy. The patient had no history of TB infection or contact with a TB patient.

**Figure 1:**
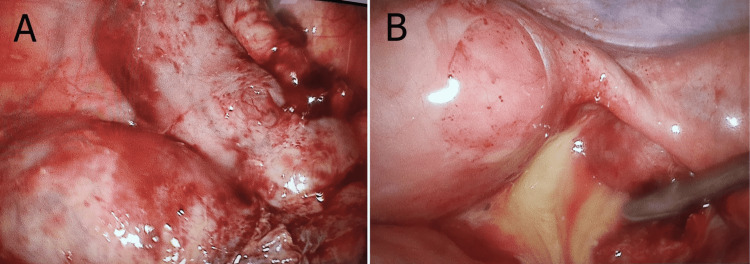
A. Laparoscopic exploration reveals two cystic masses in the right ovary with a large diameter of 4-5 cm. B. Yellowish pus indicating right pyosalpinx during laparoscopy

**Figure 2:**
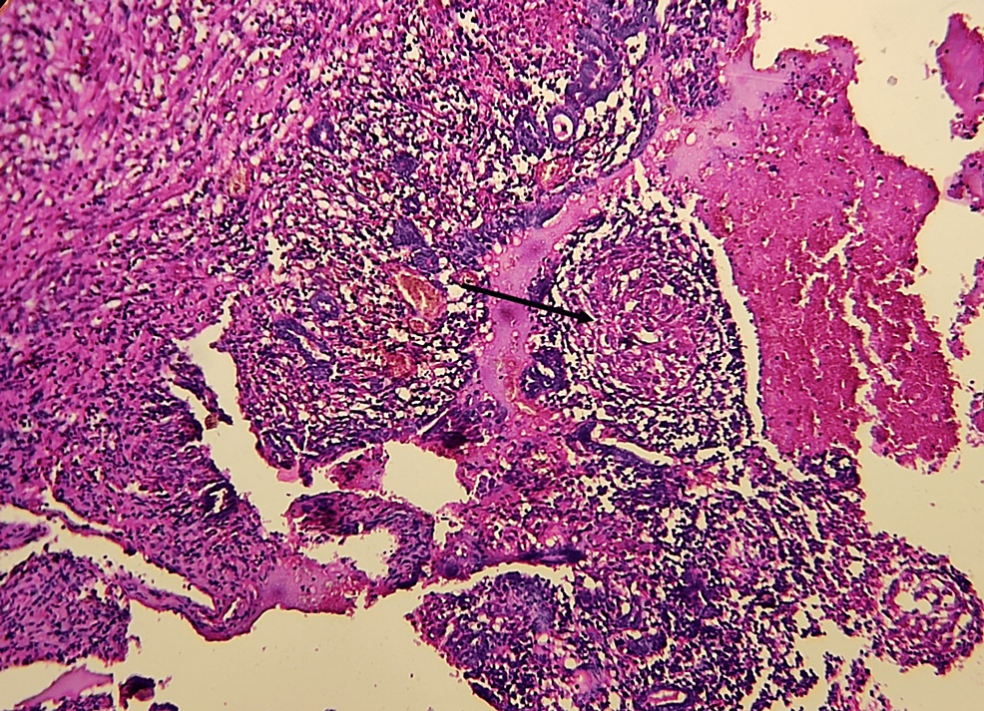
Histopathological examination showing typical giant epithelioid granulomas of tuberculosis

As part of her IVF workup, she was admitted for a routine pelvic ultrasound. She was conscious, alert, and well-oriented with normal vital parameters. Her physical examination revealed no abnormalities. The pelvic ultrasound was performed, which fortuitously revealed two cystic structures of regular margins in the right ovary with a diameter of 4-5 cm. However, the patient denied fever, night sweats, pelvic pain, or other genital or urinary signs. We decided to proceed with a diagnostic laparoscopy (Figure 1), which revealed multiple epiploic parietal adhesions causing difficult access to the rest of the genital organs. The adhesiolysis was effortful and complex. A right pyosalpinx was seen occupying the entirety of the pelvis, reaching the left appendix, and covering the right ovary. The dissection of the adnexal mass discharged yellowish pus. Right salpingectomy was performed followed by histopathological examination (Figure 2), revealing a granulomatous inflammatory process. The bacterial culture was positive for Mycobacterium tuberculosis and a diagnosis of follicular TB was made.

We started the patient on an intensive phase of anti-TB therapy (ATT) consisting of daily doses of isoniazid, rifampicin, pyrazinamide, and ethambutol, followed by a continuous phase of ATT for a total duration of six months.

## Discussion

In 2020, approximately 1.5 million people died from TB [2]. An estimated one-quarter of the world's population has TB in its "latent" form [4]. TB is considered endemic in Morocco with more than 30,000 newly diagnosed and treated cases of all forms each year [2]. The lifetime risk of contracting an infection is estimated at 5-15% for individuals with a competent immune system [4]. However, patients with immunocompromising conditions such as HIV/AIDS have a higher risk of developing the disease, particularly abdominal and pelvic TB [4,5]. Other risk factors that predispose patients to TB are low-income status, malnutrition, and limited access to medical care [4]. In our case, no risk factors for TB infection were present. An HIV serology was not performed.

TB most commonly affects the lungs - EPTB accounts for only 10% of cases [2]. Some reports suggest that female genital TB is the most common extrapulmonary localization of TB [4]. However, due to the asymptomatic forms of the disease, its different clinical presentations, and frequent incidental diagnosis, the exact incidence of genital TB cannot be determined [1,8]. The seeding of the pathogen usually occurs after puberty as the blood supply increases to the genital organs, resulting in a facilitated spread of the bacilli in the pelvic area. As a result, more pathogens reach and infect this area [1]. Genital TB infection is also associated with various chronic diseases, as well as psychological and physical stress [8].

Genital TB typically involves epididymal nodules in men and chronic salpingitis in women [2,7]. In female genital TB, the fallopian tubes are the most commonly affected organs (95-100% of cases), followed by the endometrium (50-60% of cases), ovaries (20-30% of cases), cervix (5-15% of cases), and uterus (2.5% of cases) [1,4,6,8]. The endometrium and fallopian tubes are almost always involved, and isolated ovarian infections (as in our case) are rarely described [9]. Pulmonary TB might be found prior to the ovarian disease but not always, as demonstrated by our case [9]. Women may present with various symptoms, but they can also remain asymptomatic [1,4,6]. According to some reports, 11-40% of genital TB female patients are asymptomatic and often not diagnosed until their diagnostic workup at infertility clinics [1,6]. Extensive screening for TB must be widely conducted before IVF procedures as previously rare cases of congenital TB occur when females are not initially screened due to the increasing availability of assisted reproduction technologies [10].

Genital TB is difficult to diagnose for several reasons. Firstly, genital TB is often asymptomatic, resulting in significant diagnostic delays. Second, Its presentation is similar to other genital malignancies such as ovarian carcinoma [7,8]. The definitive diagnosis of TB can only be confirmed by identifying TB on an LJ medium or by acid-fast staining [1,11]. However, UGTB frequently manifests with paucibacillary forms and, as a result, one-third of culture methods remain negative [2,11,12]. According to Namavar et al., genital TB can be confirmed based on the standard histopathological appearance with typical granuloma [13]. A variety of other tests, such as tuberculin skin test and sputum staining for Mycobacterium, are available for the diagnosis of TB, but they are usually inconclusive and strong clinical suspicion is required in most cases [1,2].

As TB is known to manifest with pelvic masses, ascites, and elevation of CA-125, physicians should consider TB with other differentials such as a fallopian tube or ovarian carcinomas, especially in epidemic areas [2,4]. However, in our case, CA-125 was not obtained. Elevated CA-125 can be found in other malignancies such as breast cancer, mesothelioma, non-Hodgkin's lymphoma, gastric cancer leiomyoma, and leiomyosarcoma of gastrointestinal origin [14]. Benign conditions that may also cause an elevation of this marker include endometriosis, pregnancy, ovulatory cycles, liver diseases, and congestive heart failure cases. However, in these cases, the elevation should not exceed 500 UI/mL [14,15]. Other than that, CA-125 can be used as a follow-up marker in genital or peritoneal TB, as its normalization was associated with a response to anti-TB treatment [4]. Human epididymis protein 4 (HE4) has shown higher specificity than CA-125 as a marker regarding benign affections [16]. A retrospective study by Zhang et al. on patients with peritoneal TB compared to those with epithelial ovarian cancer (EOC) suggests that low thresholds on both markers (151.4 pmol/L for HE4 and 563.5 U/l for CA-125) could be used to differentiate peritoneal TB from EOC [17]. However, a very common infectious disease like TB should always be considered in patients with pelvic masses or suspicion of high malignancy, especially in endemic countries, to prevent invasive surgeries and unnecessary outcomes [4].

The treatment of UGTB is essentially medical for six months, comprising quadruple therapy of isoniazid, rifampicin, ethambutol, and pyrazinamide for two months, followed by maintenance treatment with isoniazid and rifampicin for four months. Surgery is reserved for cases with compressive or fistulized masses [18]. The prognosis of pelvic TB is primarily linked to infertility, with an estimated risk of 39% among young women [2]. Therefore, early diagnosis and immediate initiation of treatment are essential for a good prognosis [14].

## Conclusions

We discussed a rare case of isolated ovarian TB, incidentally diagnosed during an IVF workup, in a 40-year-old woman with primary sterility and no specific symptoms. TB is a curable and preventable disease. However, genital TB remains a serious threat among women of reproductive age with a significant risk of infertility. Women with genital TB present with various symptoms, and this case highlights the importance of screening for TB before an IVF procedure in women with infertility, especially in epidemic countries.
